# Gremlin 1 — small protein, big impact: the multiorgan consequences of disrupted BMP antagonism^†^

**DOI:** 10.1002/path.5479

**Published:** 2020-06-20

**Authors:** Sarah Gooding, Simon J Leedham

**Affiliations:** ^1^ MRC Molecular Haematology Unit, Weatherall Institute of Molecular Medicine University of Oxford Oxford UK; ^2^ Intestinal Stem Cell Biology Laboratory, Oxford Centre for Cancer Gene Research, Wellcome Trust Centre for Human Genetics University of Oxford Oxford UK

**Keywords:** Gremlin 1, bone morphogenetic protein, stem cells, haematopoiesis

## Abstract

Highly conserved, complex and interacting morphogen signalling pathways regulate adult stem cells and control cell fate determination across numerous different organs. In homeostasis, the bone morphogenetic protein (BMP) pathway predominantly promotes cell differentiation. Localised expression of ligand sequestering BMP antagonists, such as Gremlin 1 (Grem1), necessarily restricts BMP activity within the stem cell niche and facilitate stemness and self‐renewal. In a new paper, Rowan, Jahns *et al* show that acute deletion of *Grem1* in adult mice, using a ubiquitous *ROSA26‐Cre* recombinase, induced not only severe intestinal enteropathy but also hypocellular bone marrow failure suggestive of stem cell niche collapse in both tissues. Grem1 has an increasingly recognised pleiotrophic role in a number of organ systems and is implicated across a wide range of disease states. Although the importance of Grem1 in intestinal stem cell regulation has been well described, a putative function in haematopoietic niche maintenance is novel and requires further exploration. Moreover, the complex and context‐specific regulation of Grem1, among a host of functionally convergent but structurally disparate BMP antagonists, warrants further research as we learn more about the pathogenic consequences of deranged expression of this small, but important, protein. © 2020 The Authors. *The Journal of Pathology* published by John Wiley & Sons Ltd on behalf of Pathological Society of Great Britain and Ireland.

Adult stem cells are critical to support tissue homeostasis, turnover, repair and regeneration, but cell fate determination in organ systems must be strictly regulated to prevent the unwanted development of neoplasia. A network of interacting morphogen signalling pathways, conserved across species, has evolved to regulate stem cell function. Many of these pathways generate concentration gradients through intercompartmental crosstalk, with differential expression of ligands, receptors and antagonists between different cellular components of the stem cell niche. Cell fate determination is regulated by position within these gradients. This multilevel signalling control and interactive pathway crosstalk permits amplification or attenuation of signal in a context‐dependent manner. Moreover, the dependence on intercompartmental interaction builds in capacity for functional compensation and buffering, preventing reliance on a single cell type for morphogenic control of epithelial cell fate. Unravelling the complexity of these networks, and identifying the key players within each tissue context, is critical to understanding cell fate control in homeostasis and its disruption in disease.

The bone morphogenetic protein (BMP) pathway is one of these critical morphogens, with stromal cell‐derived ligand activity promoting epithelial cell differentiation and apoptosis in multiple tissues. Thus, BMP activity must be restricted within the stem cell niche for maintenance of stemness, and this is achieved by the localised expression of a number of secreted ligand‐sequestering BMP antagonists. In the intestine, the sub‐crypt myofibroblast cells of the muscularis mucosae are the source of numerous secreted BMP antagonists, including Gremlin 1 (GREM1), Gremlin 2 (GREM2) and Chordin‐like‐2 (CHRDL2). The mechanistic effect of Grem1 is best understood predominantly through the pathological effect of aberrant epithelial expression, which promotes excessive stem/progenitor cell activity in the rare familial hereditary mixed polyposis syndrome [1]. A brief definitive report by Rowan, Jahns *et al* [2] in *The Journal of Pathology* used the ubiquitous *ROSA26‐CreERT2* recombinase, activated by oral tamoxifen, to examine the systemic impact of *Grem1* deletion on organ homeostasis in adult mice. Acute transgenic *Grem1* knockout results in the rapid development of a severe enteropathy consistent with loss of proliferating stem/progenitor cells. These findings are consistent with the hypothesised role of this BMP antagonist as a key regulator of intestinal stem cell activity [3]. However, Rowan, Jahns *et al* also demonstrated that ubiquitous knockout of *Grem1* caused the surprising and unexpected additional finding of a hypocellular bone marrow, reminiscent of aplastic anaemia [2]. This resulted in significant depletion of marrow myeloid, lymphoid and erythroid lineages, and a reduction in circulating reticulocytes [2] (see Figure 1).

**Figure 1:**
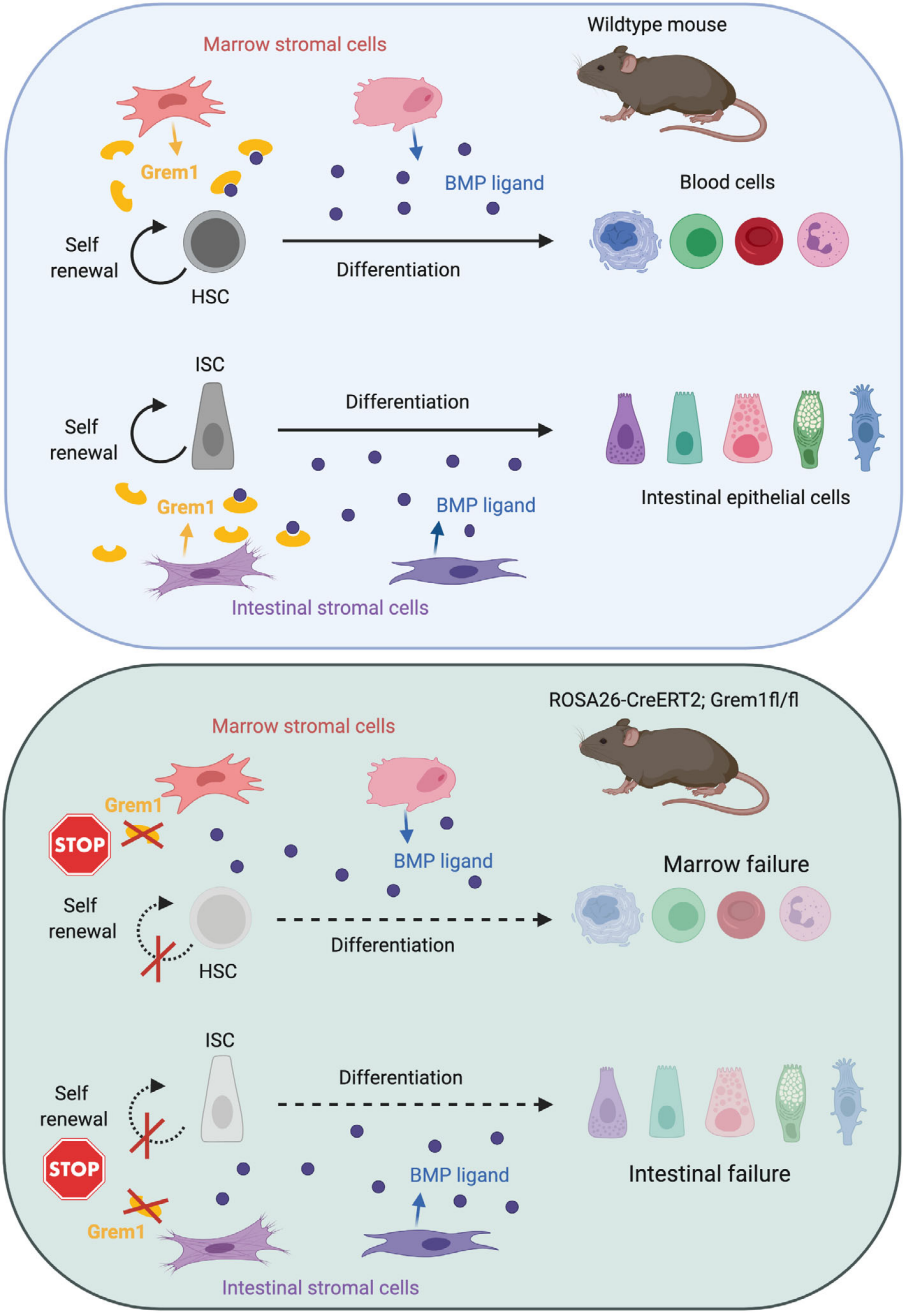
A model to show putative multiorgan impact of *Grem1* deletion. In homeostasis, localised activity of secreted BMP ligand sequestering antagonists, such as Grem1, is required to maintain adult stem cell self‐renewal, while unopposed BMP ligand activity outside of the stem cell niche promotes cell differentiation. Loss of *Grem1* through deletion using a ubiquitous *Cre* recombinase in *ROSA26‐CreERT2; Grem1* ^*fl/fl*^ mice induces intestinal and bone marrow failure, through putative failure of intestinal and HSC self‐renewal. Although the role of Grem1 in maintenance of the intestinal niche has been well studied, the possible role in the bone marrow is currently presumed, based on the findings in Rowan, Jahns *et al* [2] and requires future research for definition. ISC, intestinal stem cell.

Gradients of BMP signalling have long been known to regulate the proliferation and differentiation of haematopoietic stem cells (HSCs) in the developing embryo [4], and the critical role of BMP and Wnt signalling interplay in the commitment of bone marrow mesenchymal stem cells (MSCs) to stromal, fat or bone fate is also well described. These cells form the niche upon which HSCs depend to survive and BMP signalling disruption in this niche has been shown to disrupt functional haematopoiesis and to induce leukaemia [5]. As HSCs and their mesenchymal niche components both depend upon tightly regulated BMP pathway activity to differentiate appropriately, particularly BMP2/4 activity, the deleterious effect of ubiquitous knockout of *Grem1*, a key antagonist of BMP2/4 and 7 is of great interest. Grem1 has been shown to regulate bone marrow MSCs [6], but is not known to have an endogenous role within HSCs themselves, where BMP receptor regulation may be more relevant in fine tuning the signalling that environmental ligands and antagonists deliver. Whether the marrow failure described [2] is due to intrinsic haematopoietic lineage failure or to niche dysfunction remains to be seen, but the possibility that it is bone marrow niche‐mediated should not be discounted.

This paper [2], intimating an obligatory role for Grem1 in intestinal and HSC homeostasis, adds to a growing body of literature describing the multiorgan, pleiotropic function of this small (184 amino acid) secreted protein. In embryonic development, Grem1 interacts with Hedgehog signalling to direct mouse limb development [7]. It is also required for branching morphogenesis in the kidney and is implicated in the pathogenesis of diabetic nephropathy and kidney fibrosis [8]. In the lung, endothelial Grem1 has been implicated in vascular remodelling in pulmonary hypertension [9]. Moreover, Grem1 expression is functionally required for postnatal skeletal stem cell function [6] and marks a population of recruited MSCs in mouse models of stomach neoplasia [10]. Triangulating the data provided by Rowan, Jahns *et al* together with accumulating literature evidence implies a critical role for Grem1 in embryonic development, homeostatic adult tissue stem cell regulation, regeneration and neoplasia, and indicates a key role for this antagonist in multiorgan BMP pathway regulation.

As the pathological implications of disruption of Grem1 expression emerge, this paper highlights some key residual research questions. Rowan, Jahns *et al* [2] report an apparent obligate requirement for tonic Grem1 signalling in both intestinal and haematopoietic homeostasis. The intestinal findings are analogous to a recent publication demonstrating a similar gut phenotype following diphtheria toxin‐induced killing of *Grem1* expressing cells [11]. However, both findings contrast with the absence of intestinal phenotype when intestinal *Grem1* was deleted using a different ubiquitous *Cre* recombinase (*CAGG‐CreERT2*) [3]. The differences could be explained by variable timing, activation and cellular targeting in the different animal models used, but may also point to a wider unknown – the importance of functional redundancy and the ability of tissues to compensate for individual antagonist loss. There are numerous functionally convergent, but structurally disparate, BMP antagonists variably expressed across different tissues, often with distinct mechanisms of action. The context‐specific role, the cumulative effect, and the buffering capacity these different molecules provide is not understood, and mapping hierarchies of the different antagonists will be key to identifying the principle regulatory molecules within each tissue.

Based on the data presented here, Grem1 would appear to be a functionally important molecule in adult stem cell control, but the regulation of this gene also presents additional levels of complexity that need further research. Families with hereditary mixed polyposis syndrome have a germline 30 kb duplication on chromosome 15, upstream of the coding region of the *GREM1* gene, but patients present with a colonic neoplastic disease phenotype. This is caused by a tissue compartment switch in expression from intestinal stromal to epithelial cells, with no apparent effect in other organs [1]. In contrast, in mouse limb development models, expression of Grem1 in the developing limb bud is controlled by a cis‐regulatory region downstream of the *Grem1* locus within an intron of the *Formin* gene [12]. Thus, it seems that variable tissue expression of Grem1 is controlled by complex, context‐specific gene regulation.

From this work and the increasing interest in the field, it is becoming evident that Grem1 plays an important role in modulating BMP signalling across multiple tissues and that disruption of this intercompartmental signalling pathway contributes to disease states in numerous organs. However, the more we learn, the more we need to understand through further research before we can realise the potential therapeutic benefits of manipulation of Grem1 signalling across a range of disease states.

## Author contributions statement

SG and SJL wrote the manuscript.
